# Elevated mRNA level indicates FSIP1 promotes EMT and gastric cancer progression by regulating fibroblasts in tumor microenvironment

**DOI:** 10.1515/med-2024-0964

**Published:** 2024-05-10

**Authors:** Yao Liu, Xinju Jiang, Xiuchun Yan, Shuo Yang, Xiulan Bian, Yue Wang, Qi You, Lei Zhang

**Affiliations:** Department of Cancer Prevention and Physical Examination Center, Harbin Medical University Cancer Hospital, Harbin, 150081, P. R. China; Department of Pathology, Harbin Medical University, Harbin, 150076, P. R. China; Department of Gastroenterological Surgery, Harbin Medical University Cancer Hospital, Harbin, 150081, P. R. China; Department of Pharmacology & Toxicology, Wright State University, Dayton, 45435, United States of America

**Keywords:** gastric cancer, RNA interference, FSIP1, mRNA, fibroblasts, tumor microenvironment

## Abstract

Fiber sheath interaction protein 1 (FSIP1) plays a crucial role in cancer development and occurrence, but its influence on gastric cancer is still unclear. In this study, differential mRNA analysis was performed by TCGA database for the Limma analysis algorithm, and the gene ontology, the Kyoto Encyclopedia of Genes and Genomes, and the gene set enrichment analysis (GSEA) were used for bioinformatics functional enrichment analysis. A gastric cancer cell model with FSIP1 mRNA knockdown was constructed by RNA interference. Cell counting kit-8 and transwell migration/invasion assay were performed to verify the cell function, and western blotting was employed to confirm the expression of target genes. The GSEA analysis revealed that FSIP1 was associated with epithelial-mesenchymal transition (EMT). The high expression group also had a significant positive correlation with the markers of fibroblast in tumor microenvironment (TME). Western blotting showed that FSIP1 was generally upregulated in gastric cancer cell lines. FSIP1 mRNA knockdown cell lines inhibited gastric cells proliferation, migration, and metastasis *in vitro*, and the protein levels of EMT-related markers N-cadherin and vimentin were reduced. Our work proved that FSIP1 promoted EMT by regulating fibroblasts in the TME, thereby promoting the carcinogenic activity of cancer cells in proliferation, invasion, and migration. FSIP1 may take a role of the occurrence and could be a potential therapeutic target and offer a new insight into the underlying mechanism of gastric cancer.

## 1 Introduction

Gastric cancer is still one of the most prevalent cancers, with approximately 1,089 million new cases and approximately 768 million deaths each year [1]. In recent years, the diagnosis rate of early gastric cancer has increased to a certain extent. In Japan, approximately 50% of cases can be diagnosed early and 90% of cases can survive 5 years [2], nevertheless, due to the lack of evident characteristics of early gastric cancer, its detection rate is low, so, for most patients (>70%), gastric cancer was diagnosed in the advanced stage [3]. Therefore, it is difficult to improve the survival rate of most patients by early gastric cancer diagnosis methods.

Up to the present, the treatment methods for gastric cancer mainly include the combination of surgery, chemotherapy, molecular targeted therapy, and immunotherapy. These methods can reduce the size of the tumor and reduce the risk of recurrence and metastasis, but there are still patients with extensive metastasis and spread [4]. Even though chemotherapy improves the progression-free survival and overall survival, the median survival is usually less than 1 year in patients with advanced gastric cancer [5]. In the past decades, the incidence of gastric cancer has declined, but in most parts of the world, the overall 5-year relative survival rate of gastric cancer is only 20% [6]. The role of tumor microenvironment (TME) in tumor progression has attracted more and more clinical experts’ attention, and remarkable achievements have been made [7]. However, the mechanism of how TME promotes the occurrence and development of gastric cancer still needs more research and supplement. It is therefore of vital importance to understand the interaction and progression mechanism between gastric cancer and TME, identify effective prognostic factors, and find therapeutic targets in order to improve survival rates.

Our previous study reported that fiber sheath interaction protein 1 (FSIP1) was closely associated with gastric cancer progression and could construct a prognostic model based on expression levels. FSIP1 is a mRNA encoding protein, which is involved in the early development of spermatogonia [8]. protein, was highly expressed in a variety of cancer tissues, but not in normal tissues except testis and hypophysis [9,10,11,12]. And a few studies reported that FSIP1 may play a significant role in carcinogenicity and tumorigenesis [13,14,15,16,17]. This also indicated that FSIP1 is closely related to tumor progression. However, the exact role of FSIP1 in gastric cancer and its underlying mechanism have not been reported.

This study intends to construct FSIP1 silenced gastric cancer cell model by RNA interference technology, explore the influence of FSIP1 on the degree of malignancy of gastric cancer cell *in vitro*, and further elucidate the molecular mechanism of FSIP1 promoting the development of gastric cancer.

## 2 Materials and methods

### 2.1 Bioinformatic analysis

From the TCGA database (https://portal.gdc.cancer.gov/) we downloaded RNA-seq data from GC and normal stomach tissue. We used the “limma” package to perform differential mRNA analysis between the two groups, and removed mRNAs with a value of 0 in the expression profile dataset greater than 50%. Data calculations were performed using vomm, ImFit, and eBay methods. The different significances for each mRNA was finally obtained, in order to ensure that the results had obvious significance |log Foldchange| > 1.5 and the mRNA with false discovery rate (FDR) <0.05 was selected. For the gene ontology (GO) analysis of differential gene sets, we used the GO annotations of genes by “org.Hs.eg.db” package, respectively, showing the results of Biological process (BP), Cellular component (CC), and Molecular function (MF). For Kyoto Encyclopedia of Genes and Genomes (KEGG) analysis, we obtained the latest gene annotations of KEGG Pathway through KEGG rest API (https://www.kegg.jp/kegg/rest/keggapi.html), and finally performed enrichment analysis with “clusterProfiler” to obtain the results of gene set enrichment. The “clusterProfiler” package was also used in the gene set enrichment analysis (GSEA) to study pathways enriched in high-risk groups to investigate underlying mechanisms, the reference genome was the hallmark, and the selection condition was |normalized enrichment score| >1, nominal *P-*value <0.05 and FDR *q*-value <0.25.

### 2.2 Cell culture and cell transfection

Gastric cancer cell lines AGS, BGC-823, HGC-27, and MKN-28 cells (AGS is a medium-differentiated adenocarcinoma cell line, BGC-823 is a poorly differentiated adenocarcinoma cell line, HGC-27 is an undifferentiated adenocarcinoma cell line, and MKN-28 is a middle-differentiated tubular adenocarcinoma cell line) were purchased from Wuhan Procell Biotechnology Co., Ltd (China). In addition to the AGS cell line cultured with Ham’s F-12 complete medium (PM150810), other cells were cultured in RPMI-1640 (McCoy’s 5A medium PM150110) with 10% FBS (164210-50) and 1% penicillin-streptomycin solution (PB180120) at a CO_2_ concentration of 5% and a temperature of 37°C. In this study, we used the BGC-823 cell line to knock out FSIP1 to study the effect of FSIP1 on proliferation, migration and invasion of gastric cancer cells. Insert 20 µg lentivirus expression vector GV493 (Genechem, China) into the designed and synthesized anti-FSIP1 shRNA, together with 15 µg psPAX2 and 10 µg pMD2.G transfected 293T cells together. The shRNA sequences targeting FSIP1 are shown in Table 1. After 48 h, the cell supernatant was collected, and the concentrated lentiviral particles and polybrene were added to BGC-823 cells. When the number of infected cells was sufficient, cells were screened using different concentrations of purine.

**Table 1:** Sequences of shRNA targeting FSIP1

Name	Sequences of shRNA targeting FSIP1
FSIP1-135-F	CCGGGCAAGCAACTTGAACTCTGGTCTCGAGACCAGAGTTCAAGTTGCTTGCTTTTTG
FSIP1-135-R	AATTCAAAAAGCAAGCAACTTGAACTCTGGTCTCGAGACCAGAGTTCAAGTTGCTTGC
FSIP1-590-F	CCGGCTCAGTGTTTCATACTCAAATCTCGAGATTTGAGTATGAAACACTGAGTTTTTG
FSIP1-590-R	AATTCAAAAACTCAGTGTTTCATACTCAAATCTCGAGATTTGAGTATGAAACACTGAG

### 2.3 Western blotting

Protein samples of transfected cells FSIP1sh-1, FSIP1sh-2, and GES, AGS, BGC-823, HGC-27, and MKN-45 were prepared in RIPA buffer, and total proteins were isolated and quantified using the BCA Protein Detection Kit (Beyotime Institute of Biotechnology). The protein samples were boiled for 10 min, 20 µg sample were added to each channel of the 12% polyacrylamide gel, and after separation by SDS-PAGE electrophoresis, the proteins of different molecular weights in the gel were transferred to the PVDF membrane and sealed for 2 h using 5% skim milk powder. The protein membrane was incubated with rabbit polyclonal antibody FSIP1 (1:1,000, ABclonal, USA), rabbit monoclonal antibody N-Cadherin (1:1,000, ABclonal, USA), rabbit monoclonal antibody N-Cadherin (1:1,000, ABclonal, USA), and murine monoclonal antibody β-Tubulin (1:5,000, Proteintech, USA) overnight at 4°C. After the incubation was completed, the PVDF membrane was rinsed and the protein membrane was incubated with horseradish peroxidase-labeled secondary antibodies for 1 h at room temperature. The marker was then used (Gel Imager system, Bio-Rad, USA) to expose PVDF membranes and observed the relative expression of the analyzed proteins.

### 2.4 Cell counting kit-8 (CCK-8) assay

The effect of FSIP1 on the proliferation ability of tumor cells was assessed using CCK-8 assay. First, we transferred 4 × 10^3^ BGC-823 cells to 96-well plates. A total of 100 μL of cell suspensions were added to each well, and after culturing in a 37°C incubator for 4 h, 10 μL of CCK-8 reagent (Dalian Bergolin Biotechnology Co., Ltd, Dalian, CN) was added to each well. After incubation for 4 h, the adsorption rate of the medium at a wavelength of 450 nm was measured with a microplate reader after 2 h. The detection at the specified time (24, 48, and 72 h) was repeated. Absorbance was measured for cell viability.

### 2.5 Transwell cell migration and invasion assays

The 24-well 8 μm pore size transwell plate (CoStar, USA, #3422) was used to detect cells’ migration and invasion ability. For the migration assays, we digested and centrifuged the cells of the experimental and control groups (sh-FSIP1, sh-NC) and resuspended them with a serum-free culture medium. We then performed a cell count on the cell suspension and diluted it, and finally added 200 μL of the cell suspension to the upper chamber (Density of 8 × 10^4^ cells per 200 μL). Simultaneously, 900 μL of DMEM containing 10% serum were added to the lower chamber. After 24 h of incubation, the cells migrated to the lower surface, were fixed with crystal violet staining, and stained for 30 min. For the determination of cell invasive ability, we covered the surface of the upper chamber with Matrix (Corning, USA, #356234). The remaining steps are similar to the migration experiments. Finally, the experimental results were observed under the microscope. And we counted and photographed five randomly selected visual fields under the microscope.

### 2.6 Immunohistochemistry

Tissues from patients with gastric cancer immersed in wax and wax blocks were sectioned. They were dewaxed in xylene solution and then immersed in ethanol for gradient dehydration. The sections were then rinsed with water for 5 min, subsequently washed three times in PBS, and antigen-repaired with ethylenediaminetetraacetic acid at 120°C, pH 7.4 for 3 min, treated with 3% hydrogen peroxide for 45 min to remove endogenous peroxidase. The sections were incubated with goat serum (Boster, USA) for 1 h at room temperature to block section no-specificity. They were incubated with primary antibody (Rabbit Anti-FSIP1 antibody, Bioss, bs-8575R) at 4°C overnight and placed in a 37°C incubator for 45 min the next day, then washed with PBS and secondary antibody was added. After incubation with secondary antibodies, they were rinsed with PBS and stained with diaminobidine. Nuclei were stained with hematoxylin.

### 2.7 Statistical analysis

The data obtained from the experiments were represented by the mean value ± SD from at least three independent repetitions. SPSS 22.0 software (SPSS, USA) was used for statistical analysis. We then used a two-tailed Student’s *T*-test for between-group difference analysis. Pearson correlation analysis analyzed the correlation among multiple indexes. All bioinformatic analyses were performed using R Studio software (v4.0.2). The difference was statistically significant (*P* < 0.05).


**Ethical approval:** All plans followed were in accordance with the ethical standards of the Committee for the Responsibility of Human Subjects (institutional and national), as well as the 1964 Declaration of Helsinki and subsequent versions. This study was approved by the Ethics Committee of Harbin Medical University Cancer Hospital (Approval Number: SHGC-1029).

## 3 Results

### 3.1 Bioinformatics of FSIP1

**Figure 1:**
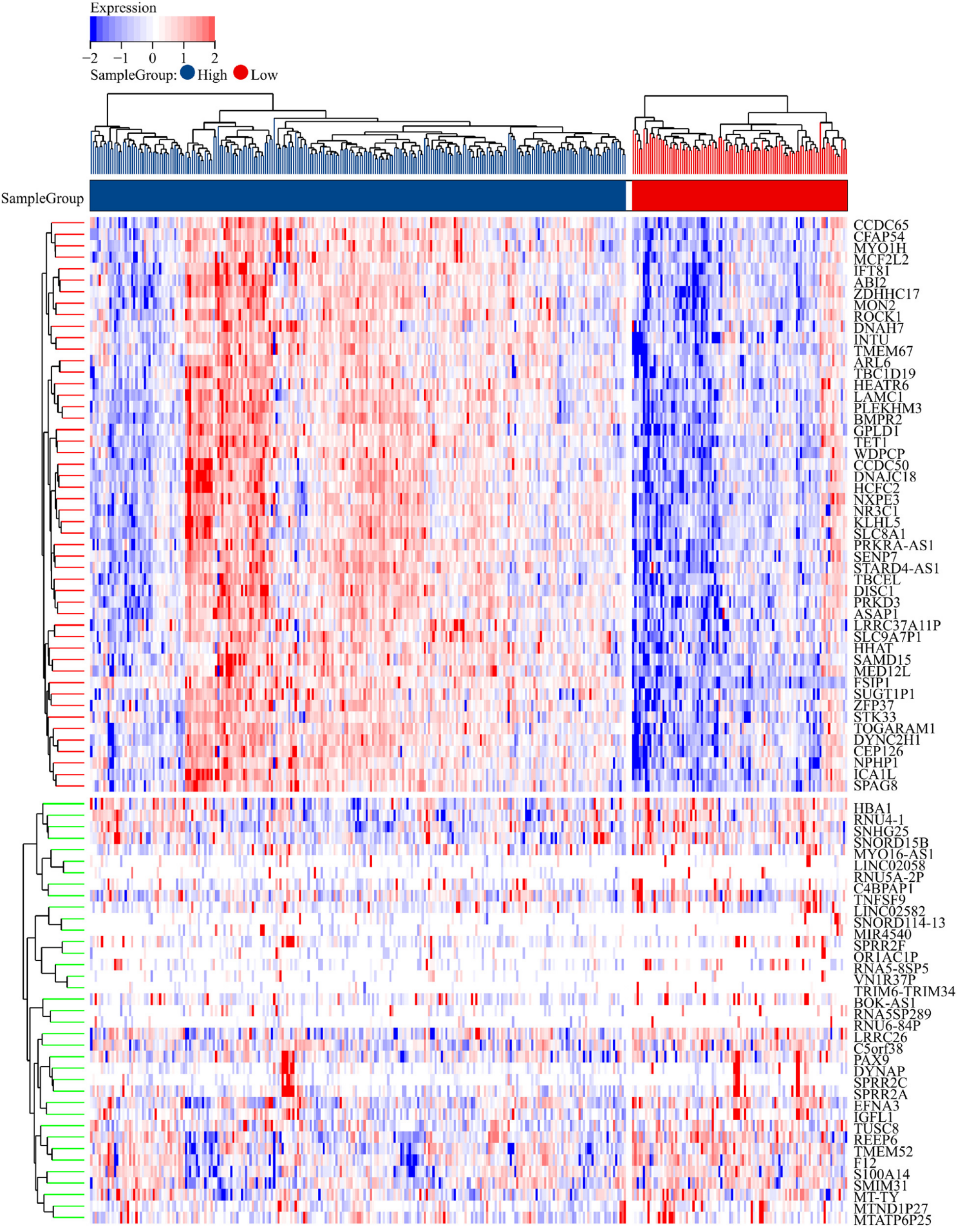
The heat map of differentially expressed genes with high and low FSIP1 expression was obtained by Limma differential analysis.

**Figure 2:**
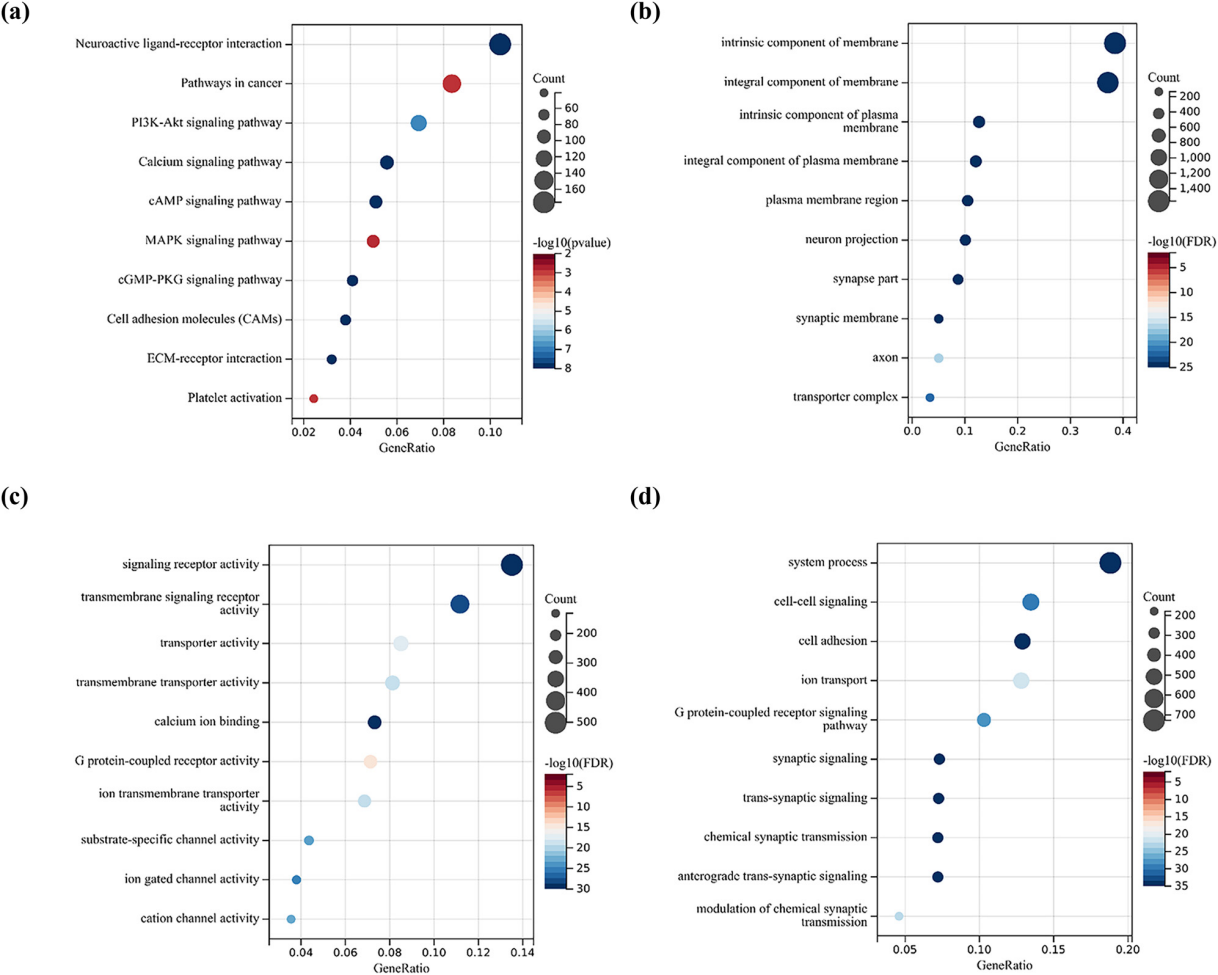
(a) KEGG functional enrichment analysis of FSIP1 and its related differential genes in TCGA dataset. (b)–(d) GO functional enrichment analysis of FSIP1 and its related differential genes revealed the main BPs involved in BP, CC, and MF.

**Figure 3:**
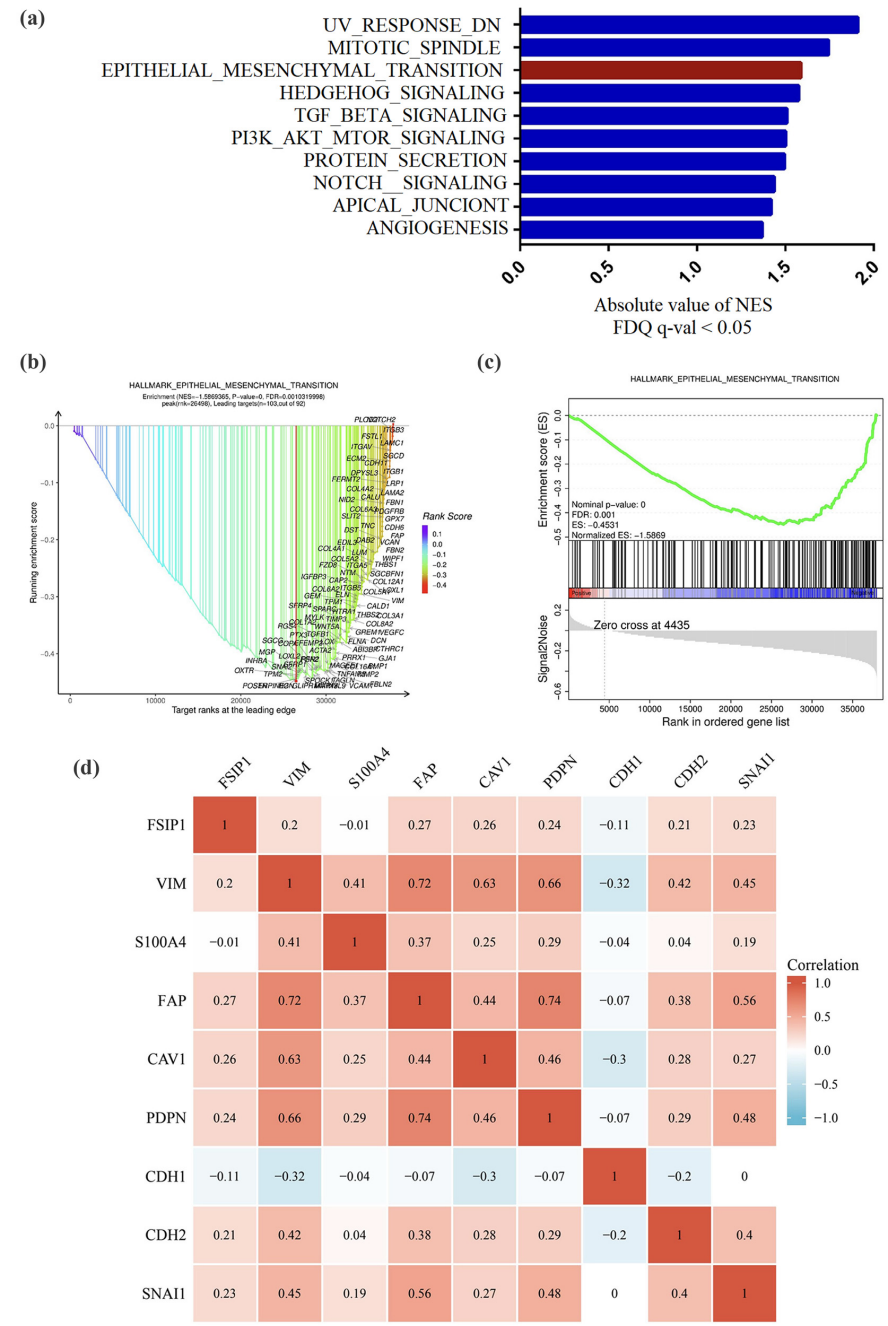
(a) and (b) The GSEA functional enrichment analysis of FSIP1. (c) The FSIP1 high-expression group was significantly correlated with EMT by the GSEA analysis. (d) The heat map of correlation between FSIP1 and fibrocyte-specific markers.

According to the disease-specific survival time of the TCGA dataset, the gastric cancer patients were divided into high and low expression groups according to the expression of FSIP1. Then, differential mRNA analysis was performed using Limma analysis method as algorithm (Figure 1), and the differential genes were analyzed by KEGG and GO functional enrichment analysis. The KEGG analysis showed that it was mainly involved in pathways in cancer, neuroactive ligand-receptor interaction, PI3K-Akt signaling pathway, calcium signaling pathway, and cAMP signaling pathway (Figure 2a). In addition, the GO analysis results showed that BP was mainly involved in system process, cell-cell signaling, cell adhesion, ion transport, G protein-coupled receptor signaling pathway, etc. (Figure 2b). In CC, it mainly participated in intrinsic component of membrane, integral component of membrane, intrinsic component of plasma membrane, integral component of plasma membrane, plasma membrane region, etc. (Figure 2c). In MF, it mainly participated in signaling receptor activity, transmembrane signaling receptor activity, transporter activity, transmembrane transporter activity and calcium ion binding (Figure 2d). The GSEA analysis showed that the high FSIP1 expression group was mainly involved in biological pathways such as UV_RESPONSE_DN pathway, mitotic spindle, epithelial mesenchymal transition (EMT), hedgehog signaling, and TGF-β signaling (Figure 3a and b). There was also a substantial correlation between the high FSIP1 expression group and EMT (Figure 3c). The expression of FSIP1 was positively correlated with fibroblasts-specific markers such as VIM, FAP, CAV1, PDPN, CDH2, and SNAI1 (Figure 3d).

### 3.2 FSIP1 is generally upregulated in gastric cancer cell lines, cell line construction (knockdown of FSIP1 in BGC by RNA interference technology)

**Figure 4:**
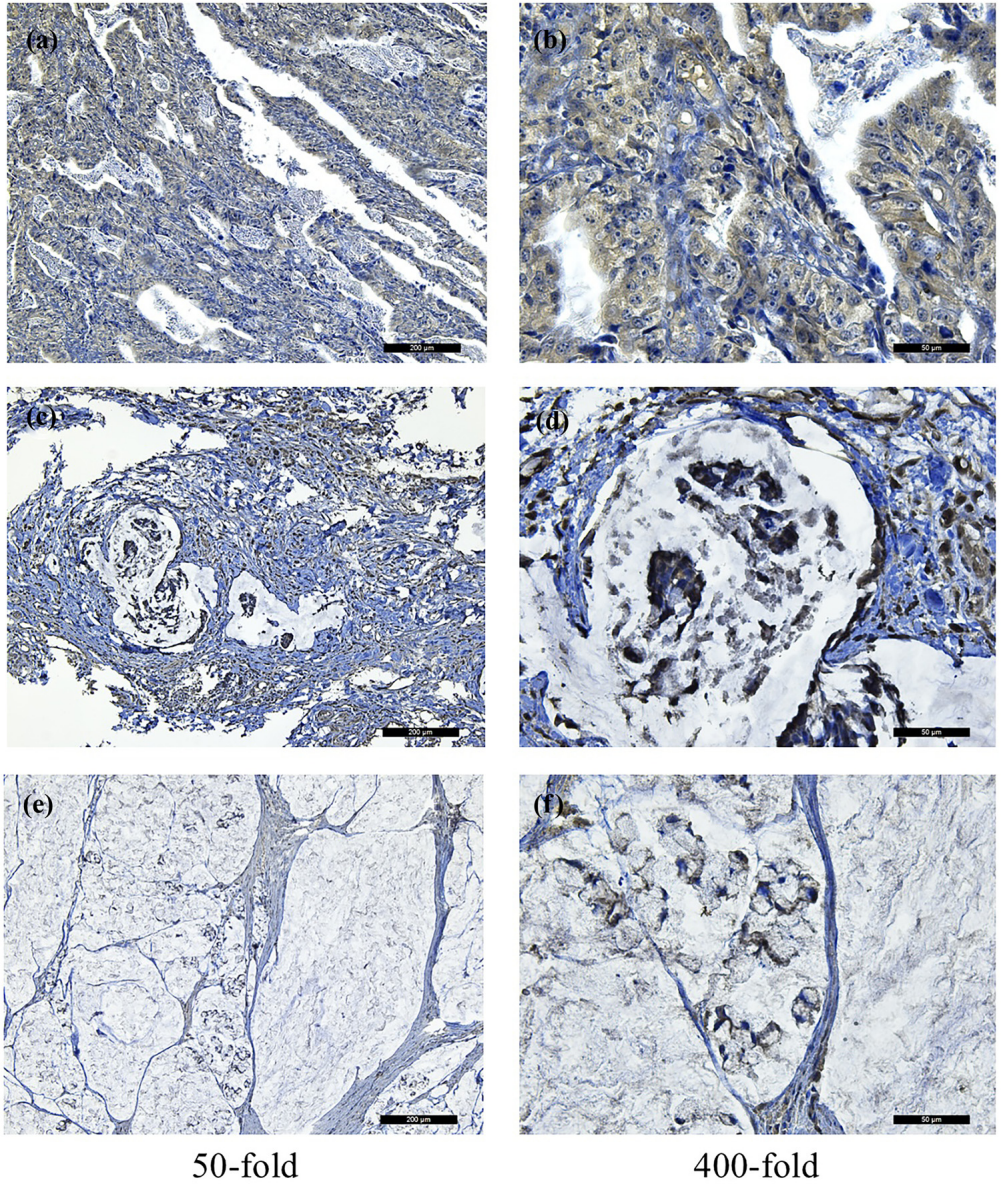
FSIP1 positive immunohistochemical expression in moderately differentiated carcinoma at 50-fold (a) and 400-fold (b). FSIP1 positive immunohistochemical expression in poorly differentiated carcinoma at 50-fold (c) and 400-fold (d). FSIP1 positive immunohistochemical expression in signet-ring cell carcinoma at 50-fold (e) and 400-fold (f).

**Figure 5:**
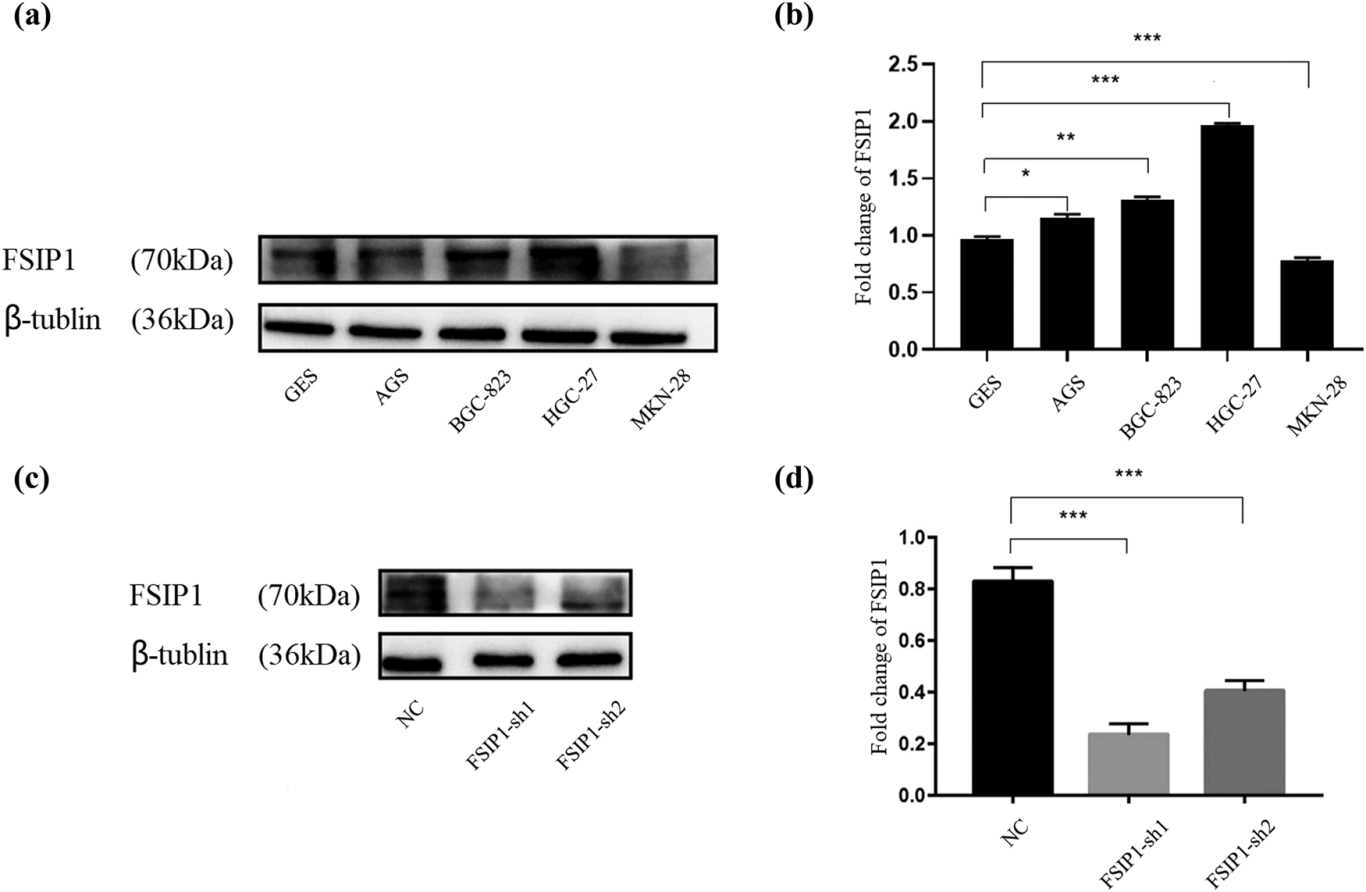
(a) Western blot showed the protein expression of FSIP1 in normal gastric epithelium and different gastric cancer cell lines. (b) Quantitative statistical analysis of FSIP1 protein expression level in normal gastric epithelium and different gastric cancer cell lines. (c) Western blot showed the knockdown of FSIP1 protein expression level in BGC-823 cell line. (d) Quantitative statistical analysis of FSIP1 protein expression level in BGC-823 transfected cell line. All experiments were repeated three times. *P < 0.05, **P < 0.01, ***P < 0.001.

We found that FSIP1 was mainly expressed on the cell membrane of gastric cancer cells. Figure 4 shows the FSIP1 positive immunohistochemical expression in moderately differentiated carcinoma, poorly differentiated carcinoma and signet-ring cell carcinoma (Figure 4a–f). We studied the expression of FSIP1 in normal gastric mucosa epithelial cell lines (GES) and gastric cancer cell lines. We obtained the expression of FSIP1 through western blot, and found that in gastric cancer cell lines, FSIP1 protein level expression was generally upregulated (Figure 5a). In addition, we constructed the BGC FSIP1 knockdown cell lines using RNA interference technology, and then we found that FSIP1 expression was significantly knockdown by western blot analysis (Figure 5b).

### 3.3 Knockdown of FSIP1 inhibits proliferation, invasion, and metastasis of gastric cancer cells

**Figure 6:**
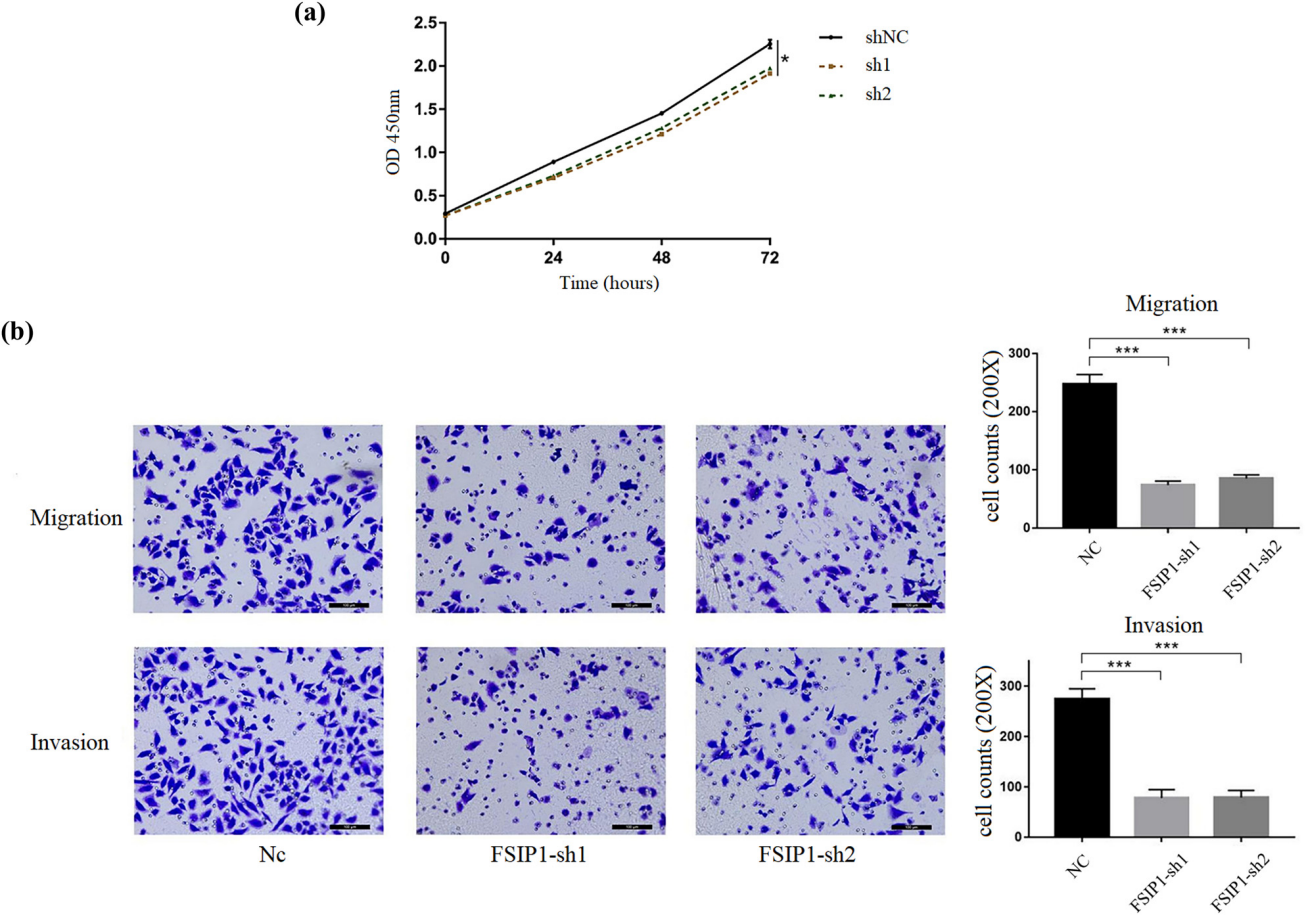
(a) CCK-8 assay showed that downregulation of FSIP1 inhibited the proliferation of gastric cancer cells in BGC-823 cell line. (b) Transwell assays showed that downregulation of FSIP1 inhibited the migration and invasion of gastric cancer cells in BGC-823 cell line. All experiments were repeated three times. *P < 0.05, **P < 0.01, ***P < 0.001.

In order to clarify whether FSIP1 is related to the growth of gastric cancer cells, we compared the proliferation ability of sh-NC and sh-FSIP1 cells by *in vitro* experiments. Downregulation of FSIP1 significantly inhibited the proliferation of gastric cancer cells, which is shown by CCK-8 assay detection and analysis (Figure 6a). To elucidate whether FSIP1 is associated with gastric cancer cells metastasis, we compared the migration and invasion abilities between sh-NC and sh-FSIP1 cells. Transwell migration assays showed that silencing of FSIP1 in BGC cell lines significantly reduced cell migration ability (Figure 6b). Furthermore, transwell invasion assay showed that the cell invasion ability of the sh-FSIP1 group was attenuated (Figure 6b). Taken together, FSIP1 can promote the proliferation, propagation, migration, and invasion of cancer cells.

### 3.4 FSIP1 functions by regulating EMT

**Figure 7:**
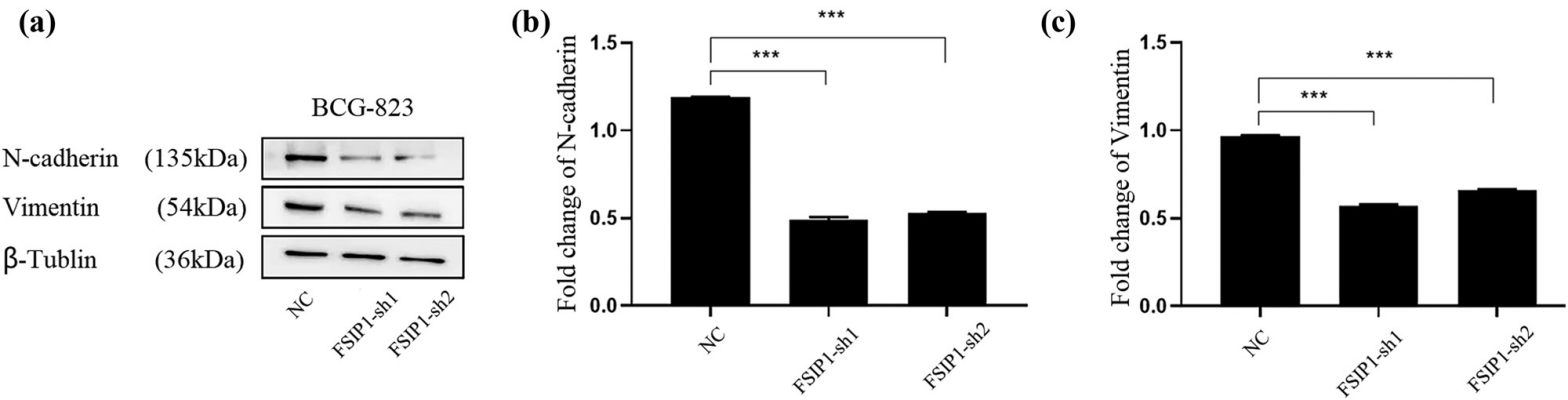
(a) Western blot showed the protein levels of N-cadherin and vimentin in NC group cells and sh-FSIP1 cells. (b) Quantitative statistical analysis of N-cadherin protein expression level in NC group cells and sh-FSIP1 cells. (c) Quantitative statistical analysis of vimentin protein expression level in NC group cells and sh-FSIP1 cell. All experiments were repeated three times. *P < 0.05, **P < 0.01, ***P < 0.001.

EMT is a crucial BP in which malignant tumor cells derived from epithelial cells acquire the ability to migrate and invade. In this study, in order to clarify whether FSIP1 is related to gastric cancer cells metastasis, we explored the effect of knockdown of FSIP1 on EMT-related markers, and we found that N-cadherin protein level and vimentin protein level were decreased in sh-FSIP1 cells (Figure 7). This suggests that FSIP1 supported the EMT of gastric cancer cells.

## 4 Discussion

Through previous studies, we established that the immunohistochemical expression level of FSIP1 in gastric cancer tissues was significantly higher than that in adjacent tissues, and the immunohistochemical expression of FSIP1 was associated with poor prognosis of gastric cancer patients. In this study, GO and KEGG analysis demonstrated that FSIP1 was related to EMT, which was an important signal pathway for tumor cell metastasis, and was involved in tumor cell proliferation and propagation. These findings were also verified in our subsequent western blot and cell invasion and migration experiments. In addition, FSIP1 was highly correlated with fibroblasts in the TME. These results also suggested that FSIP1 was closely related to gastric cancer progression and TME.

FSIP1 is a cytoskeletal structural protein of the sperm flagellin group [18]. Gamallat et al. [18] suggested that FSIP1 promotes spermatogenesis and plays an important role in acrosome biogenesis and flagellogenesis by weakening flagellar transport proteins. Some studies reported that FSIP1 may play an essential role in cell immortalization, tumorigenesis, and development. A study has found that FSIP1 can promote the proliferation and encroachment of ER+ and HER2+ breast cancer cells [19]. FSIP1 silencing inhibited the proliferation, propagation, and migration of triple-negative breast cancer cells (TNBC) *in vitro* and attenuated the growth of implanted tumors *in vivo* [20]. Liu et al. [21] showed that FSIP1 expression level may be an effective target for judging whether TNBC patients can be suitable for CDK4/6 inhibitor treatment. The activation of PI3K/AKT pathway can also be inhibited by knocking down FSIP1. And FSIP1 promoted the growth of bladder urothelial carcinoma cells by stimulating the PI3K/AKT signaling pathway, and inhibits tumor cell apoptosis [22]. These suggested to us that FSIP1 plays a major part in tumor progression. Our previous research confirmed that FSIP can serve as an effective biomarker for predicting the prognosis of gastric cancer patients [8]. The aim of this study is to explore the biological functions of FSIP1 in the progression of GC.

EMT plays a key role in tumor progression by promoting the invasion and proliferation of tumor cells and making them resistant to apoptosis [23]. Helicobacter pylori infection is a unique risk factor for gastric cancer, and some studies found that Helicobacter pylori increased EMT in gastric cancer through its cooperative network involving MMP-7, VEGF, and gastrin [24]. And some studies reported that gastric epithelial cells were co-cultured with CagA-positive Helicobacter pylori strains, and the expression of epithelial and mesenchymal cell markers were analyzed. These biomarkers of epithelial mesenchymal transition and CD44 related to tumor stem cells were up-regulated [25]. In this study, we detected that N-cadherin and vimentin were positively affected by FSIP1. These findings suggested that FSIP1 improves the metastasis of gastric cancer cells by promoting the EMT process. Our study also found a strong correlation between FSIP1 and fibroblasts. Drake et al. [26] found that carcinoma-associated fibroblasts (CAFs) were derived from tumor cells through EMT. Based on the above results, we believed that FSIP1 may promote EMT by regulating CAFs in the TME, thereby promoting gastric cancer progression.

TGF-β signaling pathway is an important pathway known to be closely related to tumor progression. In H. pylori patients, interferon-γ inhibits TGF-β1 signaling by increasing SMAD7 to downregulate the ongoing tissue damage Th1 reaction [27]. This is consistent with the tumor suppressor effect before tumor transformation. In many advanced tumors, TGF-β signaling has shifted from inhibiting cell proliferation to activating cells to undergo EMT [22]. And TGF-β1 is closely related to the invasion and metastasis of gastric cancer [28]. In this study, we showed that FSIP1 was related to the TGF-β signaling pathway by GO and KEGG analysis, which indicated that FSIP1 may regulate the development of gastric cancer by mediating or taking part in the TGF-β signaling pathway, but it was not tested in this study. We still need to explore in the follow-up research works.

In conclusion, we further studied the mRNA FSIP1 which was screened in previous studies and is associated with poor prognosis of gastric cancer. We found that FSIP1 can promote the proliferation, propagation, and migration of gastric cancer cells, and promote the occurrence of EMT by regulating fibroblasts in TME. We also noted that FSIP1 has some relationships with tumorigenesis and development-related pathways such as TGF-β. These findings indicated that FSIP1 plays vanguard roles in the progression of gastric cancer, and also suggested that the mRNA FSIP1 may become a potential target for the therapy of gastric cancer.
